# Providing Care for Older Residents With Dementia in Nursing Homes in China: Through the Lens of Adaptive Leadership

**DOI:** 10.1093/geroni/igab046.650

**Published:** 2021-12-17

**Authors:** Jing Wang, Xueli Bian, Ruth Anderson, Anna Beeber, Junqiao Wang

**Affiliations:** 1 UNC Chapel Hill, Chapel Hill, North Carolina, United States; 2 Fudan University, Shanghai, Shanghai, China (People's Republic); 3 University of North Carolina at Chapel Hill, UNC Chapel Hill, North Carolina, United States

## Abstract

This study aims to understand staff’s experiences of providing direct care for older residents with advanced dementia in long-term care facilities through the lens of Adaptive Leadership Framework for Chronic Illness (ALFCI). Semi-structured interviews were conducted with health care aides (N=35) from 2 government-owned and 2 private long-term care facilities in urban China. Directed and conventional content analysis were used, drawing upon core constructs of ALFCI. We found that health care aides are confronted with multiple challenges such as high intensity of work, stress from managing older residents’ behavioral and psychological symptoms of dementia (BPSD), a lack of access to on-the-job dementia-specific training, and a lack of support from nurses and managing team. Some of the health care aides demonstrated use of their strengths and doing adaptive work to improve work life and care for older residents by using communication cues, enhancing person-centeredness in their care, and facilitate peer interactions.

